# [Retracted] A novel role for Livin in the response to ultraviolet B radiation and pterygium development

**DOI:** 10.3892/ijmm.2026.5772

**Published:** 2026-02-24

**Authors:** Shuang-Qing Wu, Qi-Bin Xu, Wen-Yan Sheng, Lin-Ya Su, Li-Wei Zhu

Int J Mol Med 45: 1103-1111, 2020; DOI: 10.3892/ijmm.2020.4481

Following the publication of the above paper, it was drawn to the Editor's attention by a concerned reader that certain of the flow cytometric data shown in Fig. 6E on p. 1109 were strikingly similar to data that had appeared previously in a paper in the journal *Toxicology and Applied Pharmacology* written by different authors at different research institutes. In addition, a subsequent assessment of the data in the Editorial Office revealed that the cleaved caspase-3 western blot data in Fig. 4A appeared to match with the protein bands featured in a larger gel slice shown to represent the Livin data in Fig. 5A.

In view of the fact that the abovementioned data had already apparently been published prior to its submission to *International Journal of Molecular Medicine*, the Editor has decided that this paper should be retracted from the Journal. The authors were asked for an explanation to account for these concerns, but the Editorial Office did not receive a satisfactory reply. The Editor apologizes to the readership for any inconvenience caused.

